# Perceived positive social interdependence in online versus face-to-face team-based learning styles of collaborative learning: a randomized, controlled, mixed-methods study

**DOI:** 10.1186/s12909-022-03633-y

**Published:** 2022-07-23

**Authors:** Ikuo Shimizu, Yasushi Matsuyama, Robbert Duvivier, Cees van der Vleuten

**Affiliations:** 1 Center for Medical Education and Clinical Training, Shinshu University, 3-1-1 Asahi, Matsumoto, 3908621 Japan; 2 Medical Education Centre, Jichi Medical University, 3311-1 Yakushiji, Shimotsuke-shi, Tochigi Japan; 3 Center for Educational Development and Research in Health Sciences (CEDAR), University Medical Center Groningen, Antonius Deusinglaan, 19713 AV Groningen, The Netherlands; 4 Department of Educational Development and Research, Faculty of Health, Medicine and Life Sciences, Maastricht University, Universiteitssingel 60, 6229 ER Maastricht, The Netherlands

**Keywords:** Collaborative learning, Mixed methods research, Online learning, Problem-based learning, Social interdependence theory

## Abstract

**Background:**

Collaborative learning is a group learning approach in which positive social interdependence within a group is key to better learning performance and future attitudes toward team practice. Recent attempts to replace a face-to-face environment with an online one have been developed using information communication technology. However, this raises the concern that online collaborative learning (OCL) may reduce positive social interdependence. Therefore, this study aimed to compare the degree of social interdependence in OCL with face-to-face environments and clarify aspects that affect social interdependence in OCL.

**Methods:**

We conducted a crossover study comparing online and face-to-face collaborative learning environments in a clinical reasoning class using team-based learning for medical students (*n* = 124) in 2021. The participants were randomly assigned to two cohorts: Cohort A began in an online environment, while Cohort B began in a face-to-face environment. At the study’s midpoint, the two cohorts exchanged the environments as a washout. The participants completed surveys using the social interdependence in collaborative learning scale (SOCS) to measure their perceived positive social interdependence before and after the class. Changes in the mean SOCS scores were compared using paired t-tests. Qualitative data related to the characteristics of the online environment were obtained from the focus groups and coded using thematic analysis.

**Results:**

The matched-pair tests of SOCS showed significant progression between pre- and post-program scores in the online and face-to-face groups. There were no significant differences in overall SOCS scores between the two groups. Sub-analysis by subcategory showed significant improvement in boundary (discontinuities among individuals) and means interdependence (resources, roles, and tasks) in both groups, but outcome interdependence (goals and rewards) improved significantly only in the online group. Qualitative analysis revealed four major themes affecting social interdependence in OCL: communication, task-sharing process, perception of other groups, and working facilities.

**Conclusions:**

There is a difference in the communication styles of students in face-to-face and online environments, and these various influences equalize the social interdependence in a face-to-face and online environment.

## Introduction

The development of group learning in health profession education has occurred because of evidence that students in small groups exceed their counterparts in several key areas [1]. All health professionals should be able to work within their own professions [2], but should also have the ability to interact with other professionals and work as a team [3]. This attitude is beneficial not only about the healthcare outcomes for patients as well as promoting patient safety [4].

One of the most common forms of group learning is collaborative learning [5], an educational approach in which students learn while contributing meaning to their experiences and interactions with others [5, 6], such as problem-based learning (PBL) and team-based learning (TBL) [7].

In addition to the traditional face-to-face collaborative learning environment, online collaborative learning (OCL) has been developed recently [8]. Various types of OCLs have been tried with the development of information communication technology. For example, there have been several reports on OCLs, some of which succeeded in acquiring knowledge and self-directed learning attitudes [9–11]. Furthermore, OCL is used when students cannot meet simultaneously in one place because of its flexibility [12, 13].

However, simply replacing the face-to-face environment with an online one may not be sufficient to achieve the same effects as expected. One important concept that requires attention when transitioning from face-to-face is social interdependence, one of the underlying theories of collaborative learning. According to social interdependence theory, the process of structuring positive or negative interdependence is divided into three subcategories: outcome, means, and boundary [14]. Outcome interdependence is defined as an orientation toward goals and rewards. Mean interdependence includes resources, roles, and task interdependence. Resources are used by group members, some of which are utilized as joint properties. Roles are assigned to group participants, such as readers, recorders, summarizers, and encouragers. Task interdependence can be created when group members agree on how to divide and assign tasks, making each group member responsible for their learning objectives. This leads the learning group to become more productive [14]. Boundary interdependence is based on discontinuities among individuals who segregate others into separate groups. This discontinuity is created by identity (binding as an entity) and the environment (such as a working area). Positive interdependence (actions to promote the achievement of joint goals) is key to successful collaborative learning [14, 15] and is also important for health professionals in constructing relationships between other care providers [16]. This is why some medical educators aim to cultivate positive social interdependence attitudes among learners through collaborative learning approaches [16, 17].

The problem is that we have little knowledge regarding how OCLs affect social interdependence. While communication among students is essential for maintaining mutual social interdependence, attempts in OCL have been made to replace face-to-face communication. Therefore, there is a concern that the online environment reduces the effectiveness of collaborative learning [18]. However, no study has yet examined this concern. Overcoming this gap will allow us to better consider how to introduce OCLs into future curricula. In particular, if online collaborative learning is found to be non-inferior in fostering social interdependence, it could further augment online learning and increase remote participation. Conversely, if OCL is found to be inferior to face-to-face learning, some supplementary measures need to be taken in the curriculum, especially as learning teamwork is essential in medical education.

Therefore, we will conduct this study to clarify the following two points:

1. To what extent does the degree of social interdependence in OCL differ from that in face-to-face environments?

2. What aspects affect social interdependence in OCL?

## Methods

### Design and participants (Fig. 1)

This study followed a mixed-methods approach within the paradigm of pragmatism, which emphasizes solutions to research questions and integrates quantitative and qualitative research results to obtain general findings [19]. We conducted a randomized controlled trial in a crossover manner, quantitatively and qualitatively comparing online and face-to-face collaborative learning classes. The class was conducted to learn clinical reasoning in internal medicine using team-based learning (TBL) [20], a form of collaborative learning in the spring semester (April through July) of 2021.

**Fig. 1:**
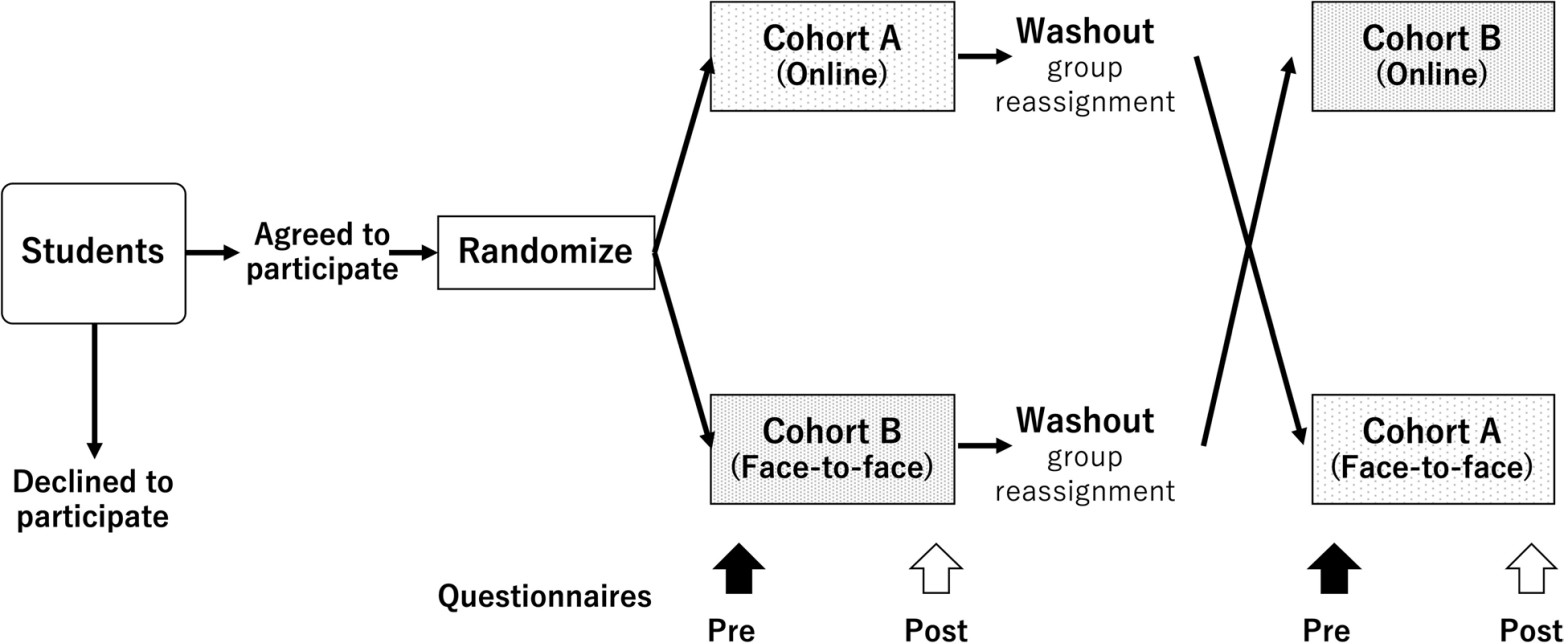
Study design

The class sequenced the learning process for the students through the following steps, based on the following standard TBL process.

*Step 1. Preparatory learning* – The students were required to complete preparatory learning assignments including reading textbooks, for the upcoming TBL session.

*Step 2. Individual readiness assurance test* – At the beginning of the class, they were asked to review their prior learning on their individual readiness assurance test (iRAT), which consisted of five single-best response multiple-choice questions about preparatory learning contents.

*Step 3. Group readiness assurance test* – Subsequently, the students were divided into groups of 7–9, and they discussed the readiness assurance test in groups (gRAT), which were the same as iRAT, and reviewed them collectively. Students were given clarification from a tutor on the concepts if they struggled during the gRAT.

*Step 4. Team application* – Finally, the students worked on tasks regarding problem solving of medical cases as a team, discussed the cases in groups, and presented their final product as a plenary discussion.

*Step 5. Plenary presentation and inter-team discussion* – Students make a presentation of their results of team application. They can appeal an answer to the one designated as “best.”

One author (IS) organized the class, and two content specialists in internal medicine served as tutors. Each class took 90–120 minutes to complete iRAT, gRAT, and group discussions.

We used Microsoft Teams (https://products.office.com/en-us/microsoft-teams/group-chat-software) to provide RATs and share case problems, and other materials were distributed electronically on Teams as a whole. Group discussion sessions were conducted face-to-face (in a large discussion room designed for TBL classes) or online (via Teams), as described below. The iRAT and gRAT scores and the content of the plenary presentations with discussions between groups were included in the summative assessment of this class.

All fourth-year students in a medical school in Japan who registered for the class (*n* = 124) were invited to participate in this research. Participants were assigned through simple randomization using Microsoft Excel to either cohort A or B. Cohort A experienced the online group sessions in the first six weeks and face-to-face in the next six weeks, followed by group reassignment within the cohort for washout; Cohort B experienced the face-to-face group sessions in the first six weeks, and online in the second six weeks, followed by group reassignment. Students who did not wish to participate in this study were excluded from the study and completed the entire class in the online environment, which was separated from the participants of the study. Those who could not participate in one of the sessions for any reason (e.g., sick leave) were excluded from the study.

### Data collection

#### Questionnaire study

To answer the first research question, we conducted a questionnaire study. All participants were asked to answer the Social Interdependence in Collaborative Learning Scale (SOCS) [21] before and after the group discussions to measure any difference in perceived social interdependence attitude. The SOCS was developed to measure students’ perceived positive social interdependence in a collaborative learning environment. It comprises of 15 items quantifying levels of three subcategories of social interdependence (outcome, means, and boundary), and its construct validity and reliability have been established [21]. We asked the participants to respond to the SOCS on a seven-point Likert scale ranging from 1 to 7 (“strongly disagree” to “strongly agree”). Participants submitted their responses anonymously. A factor analysis of SOCS was performed to explore the underlying structure of the items in this context. We used the maximum likelihood method and Promax rotation to confirm that the factor loadings were ≥ 0.4 and were theoretically consistent. The number of factors in this study was then decided based on the results of Kaiser’s eigenvalues (≥1.0). We also calculated Cronbach’s alpha to investigate the internal consistency.

We hypothesized that online discussion might achieve less positive social interdependence than face-to-face. To estimate the appropriate size of the sample, therefore, we assumed a decreased total score of 15–20% (i.e., participants in the online environment may show a score of approximately one point lower) [22].

#### Qualitative study

In addition to the questionnaire, we also conducted focus groups to answer the second research question. We asked students to participate in the research after analyzing the summative assessment of the class and conducted focus groups comprising students who were accepted as study participants. We used theoretical sampling based on the assumption that some students had favorable perceptions of collaborative learning while others may not. We tried to form a focus group with the same members as the respective TBL groups because we wanted to stimulate them to recall the interactions between the participants during their discussions. Interviews were performed in a semi-structured manner using an interview form regarding social interdependence in online and face-to-face settings. The first author (IS) conducted the interview only after all the information related to grade determination had been submitted, to avoid authority gradients and concerns about grades influencing the interview. An administrative clerk helped organize and assisted in recording the focus groups. After collecting interview data from the students, the two authors (IS and YM) found no additional meaningful codes and concluded that the data had reached saturation point to end the interview data collection.

### Analysis

#### Questionnaire study

Normality was assessed with the Shapiro-Wilk test, which showed that the data were not normally distributed. Therefore, we compared the pre- and post- scores of SOCS using the Wilcoxon singed-rank test. In addition, we compared the subtracted (post-pre) scores between the online and face-to-face groups using the Mann-Whitney U test. A *p*-value < 0.05 was considered statistically significant. The effect sizes for comparisons were also calculated using *r* values where small effect sizes ranged from 0.1 to < 0.3, medium effect sizes ranged from 0.3 to < 0.5 and large effect sizes from ≥0.5 [23]. We used SPSS 27.0 for the statistical analysis.

#### Qualitative study

From a constructivist paradigm in which “reality” is subjective and context-specific and multiple truths are constructed by and between people, qualitative data from focus groups were analyzed using inductive thematic analysis. We coded anonymized transcripts of the Japanese scripts in accordance with the six phases proposed by Braun and Clarke [24]. The initial coding was conducted by two Japanese researchers (IS and YM). IS is experienced in qualitative studies relevant to social interdependence theory. YM was chosen to conduct initial coding because he was not directly engaged in the class but had experience in multiple qualitative studies about undergraduate medical education in Japan. The transcripts were thoroughly read and analyzed using an inductive coding approach until agreement on coding was achieved through repetitive online meetings. Representative codes were translated into English after defining the themes, and a proofreading service confirmed the translation. In the coding process, we used Microsoft Excel. The other authors (RD and CvdV) contributed to producing the report through discussions.

## Results

Seventy-eight students participated (49 males and 29 females) and were divided into two cohorts; Cohort A (*n* = 40) consisted of 26 males and 14 females, and Cohort B (*n* = 38) consisted of 26 males and 12 females. There was no significant difference in the ratio of genders between the two cohorts (*p* = 0.87), and the male/female ratio was comparable to the general demographics of this medical school (1.91 in 2021).

### Questionnaire study

We obtained 75 evaluable questionnaires from those who participated in the online sessions (48 males and 27 females) and 75 from face-to-face sessions (47 males and 28 females). In the exploratory factor analysis of SOCS (Table 1), the Kaiser–Meyer–Olkin measure of sampling adequacy was 0.87, which was satisfactory. The Bartlett’s test of sphericity was significant with *p* < .001 (χ2 = 1063.04, df = 105). Kaiser’s method indicated a three-factor structure (eigenvalue = 1.15), which was consistent with the original validation study [21]. Cronbach’s alpha for each factor was satisfactory. Therefore, we adopted the original three-factor structure for SOCS in this study.

**Table 1:** Items and factor analysis of SOCS

Items	Factors		
	Means	Boundary	Outcome
My peers rely on my presence as well as my help and support.	**1.04**	−0.14	0.04
I draw conclusions from information in group discussions.	**0.66**	0.10	0.14
My peers rely on my information and advice.	**0.63**	−0.06	0.17
I incorporate the advice of others when preparing a study plan.	**0.62**	0.211	−0.23
Discussions with other members who have different opinions will improve me.	−0.23	**0.87**	0.09
I hope my learning group is superior to others.	0.22	**0.71**	−0.17
I try to share my own thoughts and materials if they are useful to other students.	0.24	**0.66**	−0.22
For me, it is important to maintain harmony within the group.	−0.05	**0.47**	0.25
I have respect for the others with whom I interact.	0.09	**0.45**	0.28
When there are different opinions, I would like to coordinate them.	0.15	**0.42**	0.11
Group members should carefully summarize each other’s arguments.	0.08	**0.41**	0.22
It is a good idea to share the tasks for more efficient group work.	−0.01	−0.20	**0.85**
I can learn important things from other students.	−0.03	0.07	**0.75**
It is a good idea for students to help one another in their studies.	0.01	0.20	**0.70**
We learn numerous important things from one another.	0.20	0.17	**0.41**
Chronbach’s Alpha (Overall 0.90)	0.84	0.86	0.81

Extraction method: Maximum likelihood estimation with Promax rotation

The mean averages and standard deviations for the SOCS subcategories are listed in Table 2. The Wilcoxon signed-rank test showed significant progression between pre- and post-class scores for the overall SOCS in both groups. Sub-analysis by subcategories showed significant improvement in boundary and means interdependence in both groups with medium to large effect sizes. Outcome interdependence improved significantly in the online group with a medium effect size. The subtracted scores (differences in post- and pre-class) between both groups in the SOCS are illustrated in Table 3. There were no significant differences in overall SOCS scores and all subcategories between online and face-to-face groups.

**Table 2:** Comparison of the pre- and post-scores of SOCS

Face-to-face (*n* = 75)				
	Mean (SD)		*p*	*r*
	pre	post		
Overall	5.42 (0.70)	5.57 (0.64)	< 0.01	0.48
Outcome	4.98 (1.04)	5.03 (1.02)	0.11	0.19
Means	5.74 (0.82)	5.95 (0.72)	< 0.01	0.43
Boundary	5.52 (0.69)	5.67 (0.63)	< 0.01	0.45
Online (*n* = 75)				
	Mean (SD)		*p*	*r*
	pre	post		
Overall	5.41 (0.59)	5.49 (0.65)	< 0.01	0.39
Outcome	4.86 (0.97)	4.96 (0.97)	0.03	0.29
Means	5.96 (0.72)	5.96 (0.72)	0.02	0.27
Boundary	5.52 (0.69)	5.67 (0.63)	0.02	0.29

*SD* standard deviation, *SOCS* social interdependence in collaborative learning scale [21]

**Table 3:** The differences of post- and pre- class scores of SOCS and comparison between face-to-face and online groups

	Face-to-face (*n* = 75) Mean (SD)	Online (*n* = 75) Mean (SD)	U	*p*	*r*
Overall	0.12 (0.26)	0.08 (0.30)	2132.0	0.44	0.03
Outcome	0.07 (0.47)	0.12 (0.45)	2309.0	0.53	0.05
Means	0.14 (0.44)	0.07 (0.49)	2306.0	0.38	0.02
Boundary	0.12 (0.27)	0.06 (0.37)	2018.5	0.24	0.09

*SD* standard deviation, *SOCS* social interdependence in collaborative learning scale [21]

### Qualitative study

Seventeen students were enrolled until saturation ultimately, comprising four focus groups in total (three to seven students per group). They consisted of ten males and seven females, showing a comparable male/female ratio to the participants (36.7%). A higher-level synthesis of the codes eventually resulted in four major themes corresponding to the second research question, “What aspects affect the social interdependence in OCL?”: 1) communication, 2) task-sharing process, 3) perception of other groups, and 4) working facilities. Representative quotes are presented below to exemplify each theme.

#### Communication

The first theme was communication during work among group members. It included subthemes of verbal communication, non-verbal communication (such as eye contact and facial expressions), and the amount of communication. The students felt that the communication process was influenced by the amount of verbal communication and the ease of non-verbal communication. The students realized that non-verbal communication was more difficult in an online environment compared to their previous experience with face-to-face group collaboration. In particular, non-verbal communication was severely impaired when students could not use the webcam.

In addition, they felt that it was challenging to communicate with multiple people simultaneously in the online environment, and thus the amount of communication within a given time was reduced. However, students tried to compensate by increasing their verbal communication in the online environment by speaking more frequently and using text messages together.

*We had to discuss things more precisely in the online environment to get the work done. On the other hand, in a face-to-face environment, if someone did not know what to do, others could immediately help.*

*When I complete something, I sometimes talk to the person next to me first to get their opinion before I talk to everyone. As this is not possible in the online, I send it on LINE (a messaging application) to my closest members and asked him for his opinions.*

#### Task-sharing process

The second theme was the task-sharing process among the group members. It included the size of working units and the discussion during the integration. The students understood the learning objectives and the overall structure of the class even in the online environment, and they did not change their learning objectives. With regard to the size of working units, students tended to work in groups in the face-to-face environment. However, in the online environment, they moved more quickly to smaller groups or individual work and then integrated their products together. Differences in the process affected the consideration of the other members’ work. In the face-to-face environment, students made progress by discussing their understanding of each other’s work in integrating the shared work. In the online environment, the integration was performed by assembling each task with minimal discussion.

*Many members would have been more comfortable discussing their work in face-to-face group work, but not on Teams.*

*When I worked online, I would just roughly divide up the work at the beginning and then start working on my own. But with face-to-face sessions, I could seek advice when I did not understand something, or ask, “What materials are you looking at?” to share that kind of information. So I thought that was different from sharing the work.*

#### Perception of other groups

The third theme was how the students perceived the progress of other groups and the content of their products. This theme included monitoring the progress of other groups and interacting with them. In the face-to-face environment, students observed other groups from time to time to know if their group work was progressing relatively well. In the online environment, however, it was difficult to observe them directly. Accordingly, using Microsoft Teams allowed them to see the contributions from other groups, and they could see their progress. Some students discussed assignments with other groups to deepen their understanding of the learning content and their relative progress. However, it was technically impossible to accomplish this in an online environment.

During the face-to-face sessions, I would go to the other groups to ask questions and try to keep track of others’ progress. I was more aware of the other groups*.*

There is virtually an isolation between the in-group and out-group on Microsoft Teams. If we had been able to see what was going on in the other groups together, we would have had additional information to help us move forward with our group work*.*

#### Working facilities

The fourth theme was working facilities, including resource sharing and group workspaces. In terms of resources, it was difficult to use textbooks and printed materials for online discussions. Meanwhile, the students were free from the need to bring their materials to the working space, as they could participate from home or other resource-rich locations. Furthermore, they also had easier access to the Internet and digital learning materials.

As for the workplace, students discussed it around their desks in the face-to-face environment, while in the online environment, they discussed it on a video-conferencing application. The ease of use of the video-conferencing application depended on the performance of their networking devices (personal computers or tablets), and students who were not proficient with the application had trouble with group work. In addition, using online cloud services to create the products facilitated their collaborative work among group members and promoted their understanding of the learning objectives.

*If we had used mainly paper or a whiteboard, as we did in the face-to-face sessions, the number of people who could write would have been limited. In this respect, it is meaningful to be able to write various opinions together from our computers.*

*Although there are differences in knowledge and viewpoints among the members, it is good to work together on a single task.*

## Discussion

We explored the differences in social interdependence resulting from collaborative learning in face-to-face and online environments using a mixed-methods research design. With regard to the first research question, we compared the results of the pre- and post-scores of SOCS. The results showed significant improvement in their overall scores during the class. There was no statistically significant difference between the two groups, confirming the non-inferiority of the online environment. The sub-analysis of each interdependence subcategory showed that means and boundary interdependence improved both in face-to-face and OCL. Outcome interdependence showed improvement only in OCL. To address the second research question, we explored the social interdependence in OCL using a qualitative method. It was found that the online environment has different characteristics from the face-to-face environment in terms of communication, task-sharing process, perception of other groups, and working facilities. We analyze these themes based on the subcategories of social interdependence theory [14].

We found that there is a difference in the communication styles of students in face-to-face and online environments. This finding confirms previous research on communication styles in online learning communities [25]. Communication is the fundamental basis of human interrelationships and hence engages the entire social interdependence. Students were aware that non-verbal communication was hindered in the online environment and compensated for it by enhancing verbal communication. Non-verbal communication is directly related to the reinforcement of the other person’s presence and affects the development of solid relationships [26, 27].

When the number of cues to understand the other person is reduced, psychological distance is farther, and communication becomes more task-oriented [28, 29]. Since the capacity for non-verbal communication is limited on communication media such as video-conferencing applications, participants were aware of the difficulty in establishing the same kind of relationship as in a face-to-face environment and tried to compensate with verbal communication. However, since this class imposed task-oriented learning in which students were required to solve the problems of a case study, it was presumed that the discussions to solve the problems and social interdependence in OCL were not affected.

Means interdependence influenced the results regarding the second theme (the task-sharing process). Students were prevented from solving tasks through discussion, resulting in less cooperation than shared, and they felt that there was less discussion during integration. However, even though the direct discussion was reduced, there was no difference in the positive social interdependence acquired, and it was still possible to complete the required tasks. The lack of a difference in outcome interdependence on the scale could explain this result.

Outcomes in a class are generally made known in advance through syllabus documents. The results suggest that outcome interdependence does not play a major role in a TBL-style collaborative learning class if the students are aware of the outcome and task of this class, which is problem-solving through group work, regardless of the environment. This result reflects the characteristic of TBL, in which all students have the minimum readiness for the discussion. The most undermining factor for positive interdependence in task-sharing is students who do not contribute to the group work (so-called *free riders*). However, in TBL, all students have the minimum prior knowledge required to achieve the outcome through group work due to prior learning. Since the online environment is oriented toward task-oriented behavior [30, 31], the students achieved positive interdependence by sharing individual tasks under the condition. This can explain the significant improvement in outcome interdependence in OCL. This characteristic of OCL may be an advantage in an outcome-based curriculum.

In terms of resources, good accessibility to various digitized learning resources is one of the advantages of learning in the online environment. OCL can also be a further advantage for positive social interdependence since students can share a single resource at the same time. Moreover, when the products are collaboratively created in the cloud, all participants can be involved in the editing process. This process is considered to improve the degree of social interdependence as observed in the quotes. Therefore, the advantages of the online environment in sharing resources and encouraging task orientation offset the disadvantages regarding means interdependence.

Regarding boundary interdependence, i.e., distinction from other groups, a video-conferencing application such as Teams allows the users to identify only the space they are working in, making it difficult to interact with groups outside of it. In addition, the online environment tends to diminish each participant’s social attributes and individuality [32]. These effects may have contributed to the difference in SOCS scores between the online and face-to-face groups.

Table 4 summarizes the above discussions and the relationship between the four themes and the three subcategories of social interdependence theory from this study. The characteristic behaviors and attitudes belonging to the four themes either promoted or hindered each subcategory, and overall improved the social interdependence of the participants.

**Table 4:** The relationship between the four themes that influence positive social interdependence in online collaborative learning and the three subcategories of social interdependence theory

Themes	Characteristics of online environment	Subcategories		
		Outcome	Means	Boundary
Communication	Strengthening verbal communication Decreasing non-verbal communication Difficulty conducting multiple communication	Increased Decreased Decreased		
Task-sharing process	Quick transition to smaller group or individual work Insufficient discussion for integration	— —	— Decreased	Decreased —
Perception of other groups	Difficulty observing and interacting with other groups	Decreased	–	Decreased
Working facilities	Use of printed materials at home Easier access of digital materials Performance limitation of devices Group work in the cloud service	— — — Increased	Increased Increased Decreased Increased	–

It has been pointed out in recent years that learning style preferences are a myth, and they do not affect learning outcomes [33]. Although face-to-face learning is often preferred for learning team collaboration, the results of this study did not provide evidence that OCLs were inferior in constructing social dependence. The preference for face-to-face environments in collaborative learning may be a new myth. From the results of this study, we estimated some reasons for the emergence of such a myth. First, the cognitive load is intensified in the online environment, as students will need to compensate for the reduction in non-verbal communication with verbal communication. Some students will need to acquire additional skills due to the gap in proficiency with digital devices. However, this does not mean that the online environment is inferior. As the number of situations where OCL is required is expected to increase in the near future [34], it is necessary to adopt strategies that can overcome the disadvantages to build social interdependence more effectively in OCL. For example, the ability to learn in a digital environment is a new academic competency [35], and we need to expand opportunities for students to acquire it.

In addition, we found that OCL has several weaknesses in terms of promoting social interdependence, typically boundary interdependence. Many video-conferencing applications are not designed to facilitate interaction between groups. On the contrary, PBL may be easier to succeed in an online environment, as past research has demonstrated [9, 10] because the group is not conscious of the differences from other groups [36]. It is also necessary to provide additional opportunities for groups to interact more closely with each other for TBL with video-conferencing applications.

There are several limiting factors in this study. First, the results of this study were observed in Japan, which has a high-context culture, meaning people communicate based on inherent understandings rather than explicit verbal explanations [37]. Verbal communication is relatively less important in a high-context culture than non-verbal communication and each other’s attributes. As mentioned above, non-verbal communication and attribute information are harder to transmit in the online environment [26]. Therefore, the students may have been more sensitive to an insufficient supply of such information. Communication in the online environment might not be as affected in a lower-context culture.

Second, we used TBL as a collaborative learning method in this study. While TBL is regarded as an entity of collaborative learning [7, 20], the specific nature of TBL might affect the results of the study. TBL follows less constructivist procedures during the discussion compared to PBL. As written above, all students have the minimum prior knowledge required for group work due to prior learning, and discussion in TBL is based on prior knowledge. The presence of the prior learning may have enhanced the outcome interdependence of the pretest.

Also, grading was another characteristic of TBL. Since students were graded on peer review of the products as well as their group contributions, they may have been fixated on finishing the task and other factors may not have had as much impact as they felt. It has been shown in a previous study that the desire to complete a task efficiently in collaborative learning influences social interdependence [38]. We cannot deny the possibility that the typical effect of this desire on both groups in this study may have masked the difference between them. However, since any learning opportunity cannot be free from the pressure of assessment in the educational curriculum, it would be virtually difficult to subtract the influence of this desire purely.

Third, this study was conducted within the context of the COVID-19 pandemic. Although we complied with government and university infection control regulations and ran it through the ethics review process, only students who accepted the face-to-face environment participated in this study. Therefore, we cannot deny the possibility that participants’ environmental preferences may have been more unbalanced. A follow-up study in the future would provide more definitive findings after the social conditions that influenced enrollment have been eliminated.

## Conclusions

This randomized control study showed no significant difference in progress in perceived positive social interdependence between online and face-to-face TBL classes. The four themes that affect social interdependence in OCL were identified (communication, task-sharing process, perception of other groups and tutors, and working facilities). Since these various influences equalize the social interdependence of the two groups, it was thought that social interdependence through collaborative learning could be constructed in the online environment. Since there are some limitations, further research in different environments will be warranted.

## Data Availability

The datasets generated and analyzed during the current study are not publicly available due to the participants’ data and anonymity but are available from the corresponding author upon written request.

## Abbreviations

gRAT: Group Readiness Assurance Test
iRAT: Individual Readiness Assurance Test
OCL: Online Collaborative Learning
PBL: Problem-Based Learning
SOCS: SOcial interdependence in Collaborative learning Scale
TBL: Team-Based Learning
